# *Lawsonella clevelandensis* as a cause of monomicrobial breast abscess: a case report

**DOI:** 10.1128/asmcr.00019-24

**Published:** 2024-12-10

**Authors:** Meghan Lindstrom, Patricia Ferrieri, Jeffrey M. Kubiak

**Affiliations:** 1 Department of Laboratory Medicine and Pathology, University of Minnesota Medical School 12269 , Minneapolis, Minnesota, USA; Pattern Bioscience, Austin, Texas, USA

**Keywords:** *Lawsonella*, acid-fast bacilli, anaerobe, case report, literature review

## Abstract

**Background:**

*Lawsonella clevelandensis* is a recently characterized fastidious anaerobic Gram-positive, partially acid-fast bacillus associated with monomicrobial abscess formation.

**Case Summary:**

This case report features a 56-year-old female who developed right breast pain and was found to have subareolar breast abscess. Aspiration of the abscess grew pinpoint to small waxy colonies within 5 days on phenylethyl alcohol agar under anaerobic conditions. The organism was maintained with sustained growth from a subculture in thioglycolate broth. Conventional identification techniques, including matrix-assisted laser desorption/ionization time-of-flight mass spectrometry, failed to identify the organism, which was confirmed as *L. clevelandensis* by 16S rDNA sequencing.

**Conclusion:**

This case marks the fifth reported case of *L. clevelandensis* as the causative organism of monomicrobial breast abscess. Additionally, this case marks the first reported instance of *L. clevelandensis* being maintained and cultivated within liquid-based growth media.

## INTRODUCTION

*Lawsonella clevelandensis* is a recently discovered bacteria predominantly associated with abscess formation in fatty soft tissues. *L. clevelandensis* was first described in a 2013 case series of four patients found to have soft tissue abscesses geographically distributed across the United States and Canada (1). Since then, almost all reported cases of *L. clevelandensis* infection involve abscess formation or infection of fatty or visceral tissues with or without association with previous surgery and/or hardware, including hepatic and abdominal abscesses, vascular graft infections, spinal and breast abscesses, and a ruptured abdominal aortic aneurysm (2–10). Although *L. clevelandensis* remains rare, infection with this organism is increasingly recognized as a causative agent of soft tissue infection worldwide (6), emphasizing its clinical relevance and the need for further characterization of this pathogen.

## CASE PRESENTATION

This case features a 56-year-old female with a complex medical history, including type 2 diabetes mellitus, end-stage renal disease, and chronic encephalopathy presenting with progressive right breast pain for ~1 week. On examination, she had a palpable mass in the subareolar region of the right breast. She was otherwise in her usual state of health without systemic symptoms, and her physical exam was otherwise unremarkable. She had no history of recent surgery or injury to the right breast. Of note, the patient had a remote history of an incision and drainage (I&D) procedure for left breast abscess 10 years prior, cultures of which had grown *Cutibacterium acnes*. Biopsies associated with the left breast lesion and follow-up 1 year after showed benign inflammatory and fibroadenomatous changes.

Due to concern for malignancy, at this presentation, the patient underwent diagnostic mammography and right breast ultrasound (US), which revealed a 6.7 cm heterogeneous fluid collection in the right breast consistent with breast abscess. Approximately 100 mL of viscous purulent fluid was aspirated and sent for microbiological analysis, including direct specimen Gram stain and aerobic, anaerobic, acid-fast, and fungal cultures.

**Fig 1:**
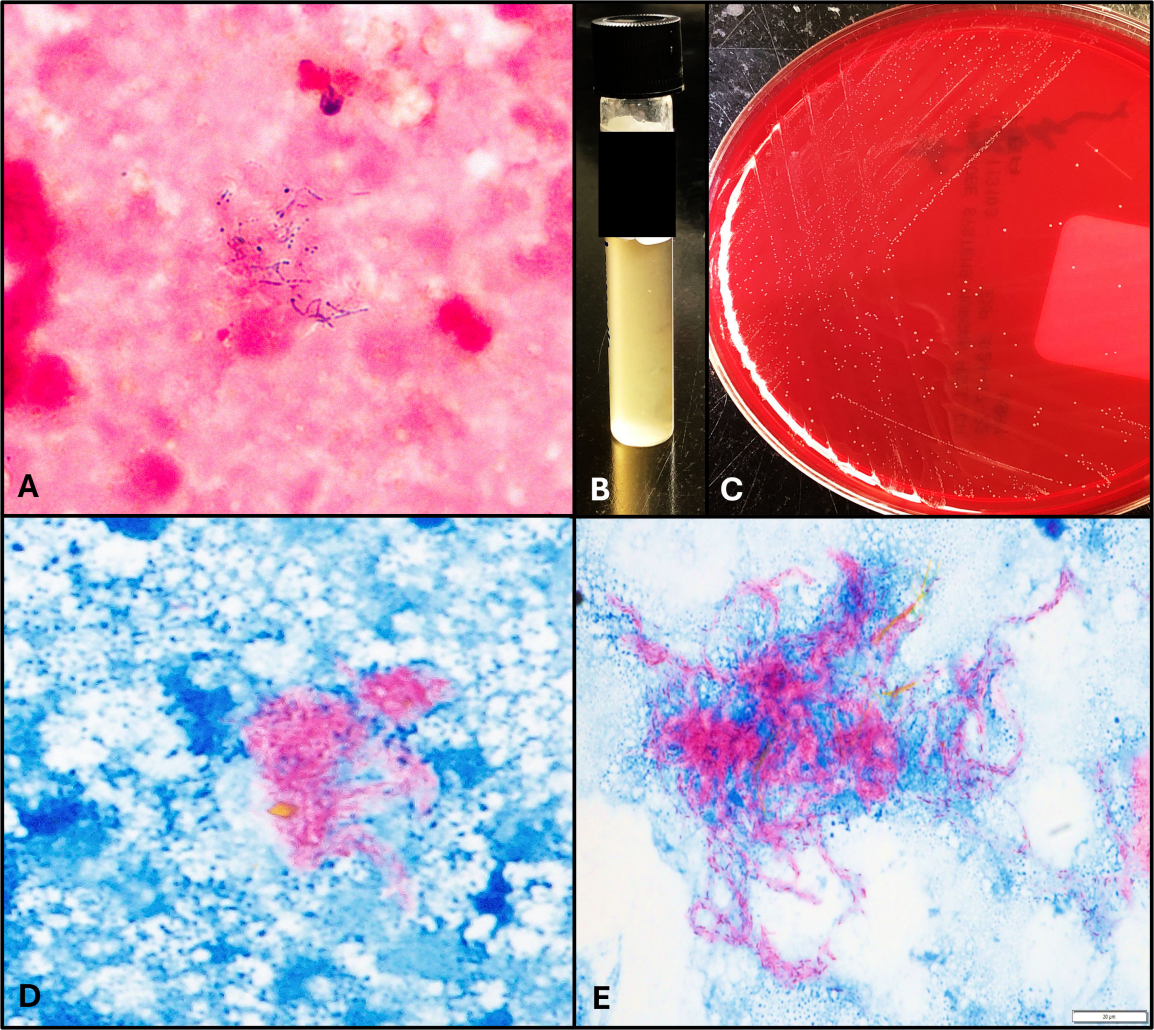
Gross and microscopic morphology of *L. clevelandensis*. The organisms were seen on Gram stain (A) and modified Kinyoun stain (D, E) from the initial abscess fluid. Pinpoint waxy colonies grew within 4–6 days on the PEA and Brucella blood agar (C), and sustained growth was achieved with subculture to thioglycolate broth (B).

The direct specimen Gram stain of the abscess fluid showed beaded Gram-positive bacilli that were revealed to be partially acid-fast bacilli (AFB) by follow-up through modified Kinyoun stain (Fig. 1A, D and E). On the fifth day, pinprick-sized waxy colonies were identified on the phenylethyl alcohol (PEA) and Brucella blood agars (both from Anaerobe Systems, Morgan Hill, CA) in anaerobic conditions confirmed as modified AFB (Fig. 1C). No growth was observed in aerobic conditions. Thioglycolate broth (Remel, Lenexa, KS) was held for extended incubation and showed growth within 21–28 days after inoculation (Fig. 1B). Colonies from a subculture of the Brucella blood agar plate could not be identified by matrix-assisted laser desorption/ionization time-of-flight (MALDI-TOF) mass spectrometry (VITEK MS, bioMérieux, Marcy l’Étoile, France); therefore, they were sent for identification via 16S rDNA sequencing by Associated Regional and University Pathologists, Inc. (ARUP) Laboratories (Salt Lake City, Utah).

While awaiting identification of the organism, the patient was started on 300 mg oral clindamycin three times daily. However, after 5 days, antibiotic therapy was stopped due to worsening encephalopathy, which was thought to be related to the initiation of clindamycin in conjunction with her underlying renal disease. At the time, the patient remained afebrile with unremarkable metabolic and hematologic studies. As she had persistent tenderness and erythema in the right breast, US imaging was repeated and identified 4.9 cm recurrent abscess, with aspiration yielding ~70 mL of purulent fluid. She subsequently underwent repeat I&D of the lesion, with the surrounding debrided tissue obtained for microbiological analysis. The Gram stains and cultures of the aspirate and the debrided tissue specimens were negative.

The patient remained hemodynamically stable and afebrile with no evidence of acute systemic illness, and her encephalopathy gradually improved. After the second I&D procedure, the patient’s right breast symptoms resolved. The 16S rDNA sequencing produced a 461 base pair product that was a 100% sequence match with *L. clevelandensis*-type strain, X1036 (NCBI reference number JX877776.1). The organism was non-viable for susceptibility testing. She was empirically started on amoxicillin–clavulanate 500 mg daily for 14 days. Seven weeks post-operatively, her right breast wound had healed without evidence of recurrent infection.

## DISCUSSION

*L. clevelandensis* is a fastidious anaerobic Gram-positive and partially acid-fast branching bacillus with occasional beaded/coccoid forms. Growth has historically been restricted to enriched solid media, such as the PEA, Brucella blood agar, and CDC blood agar, with crystalline to waxy-appearing colonies developing 4–14 days after incubation at 35°C in anaerobic conditions (1, 11, 12). No growth has been reported previously in liquid or semi-solid media, but we now demonstrate growth in thioglycolate broth within 21–28 days (11). The AFB liquid and solid, fungal, and aerobic cultures do not appear to support the growth of this organism. Other routine laboratory methods, including biochemical testing, have not been reported to reliably differentiate *Lawsonella* from other related members of *Corynebacterineae*. The MALDI-TOF databases were unable to identify *Lawsonella*, including the *in vitro* diagnostic and research use-only databases from the Bruker BioTyper, VITEK MS, and VITEK MS Prime. Phylogenetic analyses have identified *L. clevelandensis* as a member of the suborder *Corynebacterineae*, which is genetically related to other modified AFB, including the *Nocardia*, *Rhodococcus*, *Tsukamurella*, *Dietzia*, and *Gordonia* species (1, 12). *L. clevelandensis* is distinctly different from those species given its unique combination of phosphatidylglycerol and phosphatidylinositol in the absence of other phospholipids and abundant decanoic acid; the combination of fatty acids in this species may explain its predilection for fatty tissue infections (11).

This report marks the fifth documented case of *L. clevelandensis* as the causative agent of monomicrobial breast abscess (see Table 1 for a comparison of the key clinical and microbiological characteristics of the reported cases). Three out of five patients with these breast abscesses, including this patient, had overlapping comorbidities, including type 2 diabetes and obesity; one previous case occurred in a patient with a recent history of breast surgery and the other in a patient with no known risk factors (4, 13). In two of the previously reported cases, viable colonies were demonstrated on solid anaerobic media cultures, while cultures failed to grow in the other two cases (1, 4, 13). In all cases, 16S rDNA or next-generation sequencing was required to confirm the diagnosis (1, 4, 13).

**TABLE 1:** Comparisons of the key features of the reported cases of breast abscesses associated with *L. clevelandensis*

Age (years)/ gender	Comorbidities/ surgeries	Site of infection	Location	Culture growth	Method of detection	Surgical intervention	Antibiotic therapies	Outcome
44/female (6)	· Remote left breast abscess · Type 2 diabetes mellitus · Obesity · Hepatic steatosis	Left breast	Winnipeg, Manitoba, Canada	Growth on solid anaerobic media, Day 5	16S rDNA sequencing	Incision and drainage	· Vancomycin and cloxacillin · Amoxicillin clavulanate	· Resolution post-I&D without recurrence
23/female (6)	·Type 2 diabetes mellitus · Recurrent diabetic foot ulcers and osteomyelitis · Recurrent furunculosis · Remote superficial left breast abscess	Left breast	Winnipeg, Manitoba, Canada	Growth on solid anaerobic media, Day 4	16S rDNA sequencing	Incision and drainage	· Ciprofloxacin and metronidazole pre-I&D · Sulfamethoxazole– trimethoprim post-I&D	· Resolution post-I&D without recurrence
29/female (14)	None	Right breast	Lisbon, Portugal	No growth	Molecular testing (not specified)	Incision and drainage	· Sulfamethoxazole–trimethoprim pre-I&D · Amoxicillin clavulanate post-I&D	· Recurrence prior to I&D · Resolution without recurrence post-I&D
29/female (2)	Right breast augmentation with autologous fat pad injection (from the right knee)	Right breast and right knee soft tissue infection (fat pad graft)	Shanghai, China	No growth	NGS testing (not specified)	· Incision and drainage, multiple · Resection/revision of the surrounding infected tissue, multiple	· Piperacillin tazobactam pre-I&D · Cefoxitin and ornidazole post-I&D	· Recurrence post-I&D with multiple procedures required
56/female (this report)	· Type 2 diabetes mellitus · ESRD · Obesity · Remote left breast abscess · Sickle cell trait	Right breast	Minnesota, United States	· Growth on solid anaerobic media, Day 4 (initial aspirate) · Cultures of recurrent abscess showed no growth	16S rDNA sequencing	· Ultrasound guided aspiration · Incision and drainage with tissue debridement	· Clindamycin pre-I&D · Amoxicillin clavulanate post-I&D	· Recurrence prior to I&D · Resolution without recurrence post-I&D

The reported age range of patients affected by *L. clevelandensis* infection is broad, with the youngest affected patient being 2 years old and the oldest patient being 81 years old (6, 9, 10). *L. clevelandensis* infections are usually responsive to source control and administration of relatively narrow antibiotic regimens, including penicillin-based therapies (7, 8). Source control is generally achieved by I&D of the infected lesion with or without resection of the surrounding tissue (1, 3–6, 9, 10, 13). In patients affected by vascular graft of hardware infections, graft or hardware removal and/or replacement is also considered (6, 9, 10). Strains of *L. clevelandensis* were successfully cultured for susceptibility testing in 2019, showing low minimal inhibitory concentrations (MICs) associated with amoxicillin–clavulanate, ciprofloxacin, levofloxacin, metronidazole, vancomycin, clindamycin, sulfamethoxazole-trimethoprim, cefuroxime, ertapenem, and tigecycline (14).

The patient in this report initially received antibiotic therapy alone but eventually developed a recurrent abscess, ultimately necessitating I&D with debridement of the surrounding tissue for definitive management, a clinical course similar to a 2018 case (4). In this current report, it is possible that the incomplete resolution of this abscess was due to early cessation of clindamycin.

This case represents the first report in which *L. clevelandensis* has shown maintenance, or even growth in thioglycolate broth. The thioglycolate broth inoculated on the day of the specimen collection showed progressive turbidity after 21–28 days. Inoculation of plates from the thioglycolate broth showed colony growth on the PEA within 6–14 days under anaerobic conditions, while the Brucella blood agar required a minimum of 21 days. Maintenance of growth in thioglycolate broth is important for long-term passage and storage of this organism for research and other clinical purposes.

As *L. clevelandensis* is known to colonize the infundibulum of hair follicles in the fatty dermis or subdermis, the location of the patient’s abscess within the fatty breast tissue near the hair-bearing skin provides a probable route for infection (7, 15). It is also possible that the patient may have had *L. clevelandensis* associated with the previous left breast abscess 10 years prior, as cultures obtained at that time may not have been incubated in adequate conditions or for the length of time necessary to detect *L. clevelandensis*. Unfortunately, the material from the left breast abscess was not available for review for molecular studies at the time of this report. If the patient was previously infected with this organism, it is possible that the organism could have traveled by lymphatics or other means to the right breast (16).

In summary, *L. clevelandensis* is a recently described fastidious anaerobic, Gram-positive, and partially acid-fast bacterium that is associated with abscess formation in the soft or visceral tissues more often in patients with underlying comorbidities or surgical procedures. Identification of the organism is challenging and often requires molecular studies for confirmation, although the observation of Gram-positive to Gram-variable beaded rods that are partially acid-fast may prompt a consideration of the organism in differential diagnosis, particularly in otherwise culture-negative aspirates. With the increasing recognition of *L. clevelandensis* infections in patients around the world, further exploration and characterization of pathogenic mechanisms and virulence factors of this clinically significant organism are desirable.
